# Charting the structure-sequence landscape of light chain amyloids

**DOI:** 10.1093/bioinformatics/btaf167

**Published:** 2025-04-12

**Authors:** Gabriele Orlando, Rodrigo Gallardo, Alicia Colla, Joost Schymkowitz, Frederic Rousseau

**Affiliations:** Switch Laboratory, Department of Cellular and Molecular Medicine, KU Leuven, Leuven, 3000, Belgium; VIB Center for Brain and Disease Research, Leuven, 3000, Belgium; Switch Laboratory, Department of Cellular and Molecular Medicine, KU Leuven, Leuven, 3000, Belgium; VIB Center for Brain and Disease Research, Leuven, 3000, Belgium; Switch Laboratory, Department of Cellular and Molecular Medicine, KU Leuven, Leuven, 3000, Belgium; VIB Center for Brain and Disease Research, Leuven, 3000, Belgium; Switch Laboratory, Department of Cellular and Molecular Medicine, KU Leuven, Leuven, 3000, Belgium; VIB Center for Brain and Disease Research, Leuven, 3000, Belgium; Switch Laboratory, Department of Cellular and Molecular Medicine, KU Leuven, Leuven, 3000, Belgium; VIB Center for Brain and Disease Research, Leuven, 3000, Belgium

## Abstract

**Motivation:**

Light chain amyloidosis is a disease where misfolded antibody light chains (LCs) form toxic amyloid fibrils, leading to organ damage. Although LC overproduction occurs in all cases, only certain individuals develop the disease, suggesting that specific LC sequences and properties drive amyloid formation. This process is complex, involving both protein sequence and environmental factors, but mutations that destabilize the LC fold are linked to amyloid aggregation. Despite the significance of the disease, our understanding of LC fibril formation remains limited due to the lack of extensive data and technical challenges in studying amyloid structures. To address this, a tool is needed to compare unknown LC sequences with known structures and predict which amyloids are likely to adopt new conformations, guiding experimental investigations.

**Results:**

HMMSTUFF addresses this by using a Hidden Markov Model to generate similarity scores between LC sequences and existing PDB templates, eventually modeling the LC amyloid structures similar enough to known templates. HMMSTUFF on one side expands our understanding of LC amyloid fibril conformations, and on the other highlights the gaps in our current knowledge of LC structural space.

**Availability and implementation:**

HMMSTUFF is available as pypi package and as source code at https://github.com/grogdrinker/hmmstuff.

## 1 Introduction

Light chain (LC) amyloidosis is a systemic disease characterized by the overproduction and subsequent misfolding of antibody LCs (Paissoni *et al.* 2024). These misfolded LCs aggregate into toxic, insoluble amyloid fibrils that deposit in various organs and tissues, leading to organ dysfunction and ultimately death (Paissoni *et al.* 2024). The medical impact of LC amyloidosis is significant, with heart involvement being the major risk factor for mortality (Swuec *et al.* 2019).

While all LC amyloidosis cases involve the overproduction of LCs, only a subset of individuals with this condition, such as those with multiple myeloma, develop the disease. This suggests that specific sequence and biophysical properties contribute to LC amyloidogenicity and therefore to the development of the disease.

The biophysical basis of LC amyloid formation is complex, involving an interplay of factors including both the protein sequence and environmental influences, such as pH, temperature, and the presence of cofactors like glycosaminoglycans (Grigolato and Arosio 2021, Paissoni *et al.* 2024). LCs are typically found as soluble dimers in their native state, but certain conditions can trigger their misfolding and aggregation into insoluble amyloid fibrils (Rottenaicher *et al.* 2021). A hallmark of AL amyloidosis is the presence of patient-specific mutations in the variable domain (VL) of LCs, which are often linked to decreased thermodynamic stability and increased conformational dynamics, making them prone to aggregation (Rottenaicher *et al.* 2021, Paissoni *et al.* 2024). This destabilization can disrupt the delicate balance required for antibody maturation, which normally allows for antigen recognition without compromising protein stability (Otzen 2021). While the VL domain is often implicated as the primary driver of amyloidogenicity, recent studies have shown that the constant domain can also contribute to amyloid formation, and amyloid deposits can consist of various LC proteoforms, including full-length LCs (Lavatelli *et al.* 2020, Paissoni *et al.* 2024).

Despite the significance of these molecules, our understanding of how these proteins reorganize into fibrils remains quite limited. Studying fibril complexes presents several technical challenges, particularly in purification and crystallization. The few Cryo-EM structures of LC amyloids that are available are incomplete and do not represent the full diversity of LC sequences capable of forming amyloids. To make the most of the limited data, it would be valuable to develop a tool that can: (i) identify the most similar known structure to a specific amyloid LC based solely on its sequence, and (ii) predict which LC amyloids are likely to adopt conformations that differ significantly from currently known structures. Such a tool would enable scientists to explore the sequence space more systematically, maximizing the information gained from future experiments.

This paper introduces HMMSTUFF, a tool designed to address the challenges of studying LC amyloids. HMMSTUFF uses a Hidden Markov Model (HMM) to generate a similarity score between input LC amyloid sequences and available PDB templates. Additionally, it provides an alignment that can be used to model the atomic positions of each residue, facilitating structural predictions and expanding our understanding of amyloid fibril formation.

## 2 Materials and methods

### 2.1 Pipeline

HMMSTUFF is a method designed to model the structure of LC amyloids using the limited known structures currently available. Given the relatively nascent stage of LC amyloid research, the number of available LC structures is quite restricted. HMMSTUFF addresses this challenge by taking an LC sequence that forms amyloids as input and identifying the closest matching structural template from the available data.

The architecture of HMMSTUFF is specifically tailored to exploit the typical characteristics of amyloid LCs, as we did with LLPS in one of our previous works (Orlando *et al.* 2019), particularly the alternance between highly variable complementarity-determining regions (CDRs) and the more conserved constant regions. The model permits gaps only at the beginning and end of the sequence, as well as within the CDRs. This design choice is based on the assumption that insertions or deletions are rare in the constant regions. While this assumption may not always hold true, it allows for a heavily constrained alignment process, significantly reducing the likelihood of misalignments, a critical consideration given the limited data.

Each structural template is associated with its own HMM model, which provides a similarity score indicating how closely the input LC sequence aligns with the available templates. For a detailed explanation of the HMM architecture and how it functions, refer to the Methods section. The ability of HMMs to natively estimate whether a sequence is generated by a specific model enables HMMSTUFF to offer a robust similarity scoring mechanism.

As mentioned above, amyloid conformations are likely to vary significantly with different sequences. With HMMSTUFF, our goal is to identify sequences for which we can reasonably predict amyloid conformation by homology. This allows researchers to both estimate the structure of the amyloids they are studying and identify sequences that are most in need of experimental investigation due to a lack of known structural homologs. To model the structure of an input amyloid sequence, we first use HMMSTUFF to find the best matching template and assign a confidence score indicating whether the template is suitable for homology modeling. If a reliable template is found, we use its backbone structure and model the side chains with the FoldX (Schymkowitz *et al.* 2005) force field.

### 2.2 Collection of known amyloid-prone LC sequences

In addition to the conformational space, the sequence space of amyloidogenic LC is also largely unexplored. Additionally, the sequences available in public databases, such as ALBase, are often annotated with varying reliability or consist of partial fragments rather than complete LC sequences. To bridge this gap, we compiled a dataset of 254 amyloid-prone LC sequences which were experimentally confirmed to be found in LC amyloid patients and obtained through extensive literature mining. This dataset was then analyzed using HMMSTUFF and it is available in the git repository. Our dataset is composed of sequences found on NCBI genbank which were reported in literature to be amyloidogenic in patients (Prokaeva *et al.* 2007, Bodi *et al.* 2009, Klimtchuk *et al.* 2017, Swuec *et al.* 2019, Schreiner *et al.* 2024) or submitted directly to genbank by the Boston University Amyloidosis center. Additionally, the LC sequences were selected on their sequence length to only include the full length LCs which consist of at least 211 amino acids. All sequences were obtained from patients with biopsy-proven AL amyloidosis and sequenced in the Boston University Amyloidosis Center or Amyloidosis Center of the University Hospital in Heidelberg. The final dataset consists of 257 amyloidogenic LC sequences. From these 257 sequences, 25.3% belong to the kappa isotype and 74.71% belong to the lambda isotype. More specifically, the majority of the sequences are derived from the κ1 (22.17%), λ6 (21.01%), λ3 (18.29%), and λ2 (15.18%) VL germline. The other remaining sequences belong to the germlines κ3 (0.78%) and κ4 (2.33%), germlines λ4,5,7–10 were not represented in this dataset as well as κ2, 5 and 6.

We also created an additional dataset built from the AliBase database. It has been built simply using the online searching tool of the AL-Base database, created by the Boston university amyloidosis center. We selected all the sequences with “clinical category” annotated as AL-PCD, which include sequences from variable domain to full length LCs. The final dataset consists of 889 sequences.

### 2.3 Hidden Markov model structure

The first step of HMMSTUFF involves constructing a Hidden Markov Model that ensures alignments of input sequences avoid gaps in the constant regions of the LC, allowing gaps only in the complementarity-determining regions (CDRs) or within the missing segments of the structure. Each template position is encoded by a position-specific match state (M), while missing parts in the PDB template are treated as deletions. Two match segments are therefore linked by a deletion state (D). CDRs, which vary greatly in length, are represented using standard HMM profiles with match (M), deletion (D), and insertion (I) states, similar to approaches used in tools like HMMer.

Because HMMSTUFF does not generate new backbone structures, this model structure is designed to minimize misalignments in the conserved constant region of the LC, in which gaps could otherwise compromise the tool's ability to model amyloid structures. The model is therefore specifically optimized for amyloid light chains (AL).

**Figure 1.**
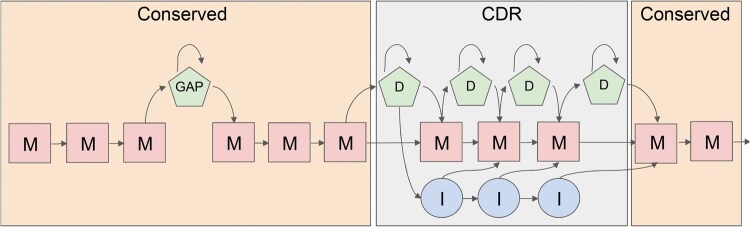
Structure of the HMM to define similarity scores in HMMSTUFF. In the conserved regions of the LC, insertions and deletions are not allowed to reduce the number of parameters and limit the chance of small misalignments that could impair HMMSTUFF’s ability to model LC structures. Gaps are only permitted where there is a missing structural segment in the template PDB. The CDR is modeled as a standard HMM profile, allowing both insertions and deletions.

A separate HMM is built for each template, and the sequence score for each of them is calculated as the ratio between the backward probability of the input sequence and the average backward probability of 100 random shuffles of the sequence. This method helps normalize scores for sequence length and amino acid composition. The HMM can output an alignment of the input sequence with the template using the Viterbi algorithm. The HMM structure is shown in Fig. 1.

### 2.4 Hidden Markov model training

HMMs have two key types of learnable parameters: emissions of each state and transitions between hidden states. Initially, we assigned dummy values to these parameters and then refined them using our dataset of amyloid light chain (AL) sequences.

For emissions, we set a 0.8 probability for a match and 0.2 for a mismatch, distributed equally across the 19 other amino acids in each match state. In deletion states, each amino acid was given an equal probability of 0.05.

For transitions, within the CDRs, the probability of opening a gap was set at 0.1 (0.05 for transitioning from match to deletion state, and 0.05 for match to insertion), with a 0.9 probability of extending the gap. Outside the CDRs, match states form a chain connected by deletion states whenever there are missing residues in the template. These deletion states have a 0.9 probability of being extended and a 0.1 probability of terminating.

This initial dummy setup was then optimized using the Baum-Welch algorithm, refining the parameters to better model the LC amyloids. For each model, we followed a multi-step approach. First, we scored all sequences in the dataset using the initial model, selecting the top 30% of sequences to retrain the HMM via Baum-Welch (implemented in Pomegranate) with inertia set to 0.9 and a stop threshold of 50. This tuned model was then used to rescore the dataset, selecting the top 20% for further retraining. The process was repeated once more with the top 10% to produce the final model.

Finally, we used this model to score the dataset and trained a quantile transformer from scikit-learn library to scale the scores between 0 and 1, making them easier to interpret.

### 2.5 Template identification

To identify the best template for an input sequence, we score the sequence against all six trained models, collecting both the score and the corresponding alignment. Structural modeling is restricted to gapless segments of the light chain amyloid (LA). We consider a structure to be reliably modelable only when one of the template scores exceeds 0.5 (above the 50th percentile) and at least 80% of the sequence aligns to the template without gaps.

### 2.6 Generation of the LC amyloid structure

Once a suitable template is identified, the corresponding portion of the LA is modeled based on the selected template. Any part of the template backbone not present in the input sequence is pruned using a Biopython script. The side chains are then modeled using FoldX's buildmodel command, while the backbone conformation remains unchanged, ensuring that only the side chains are adjusted to fit the input sequence.

### 2.7 Data analysis

**Figure 2.**
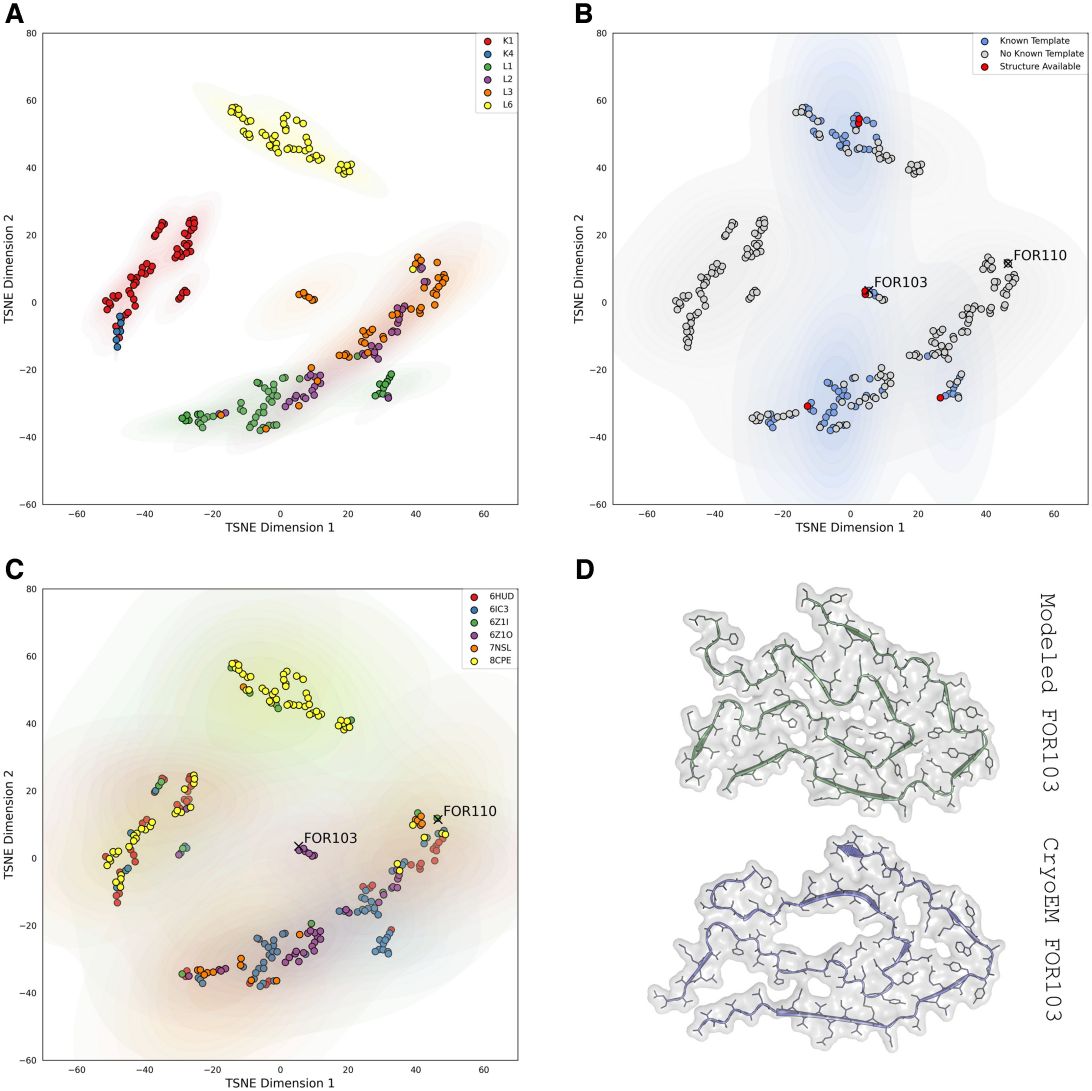
Analysis of known LC amyloid sequences using HMMSTUFF. The TSNE plots represent the distribution of sequences based on the scores assigned by HMMSTUFF. In (A), data points are color-coded according to their germline classification, showing distinct clustering patterns, with Lambda VL6 forming a separate cluster. (B) Highlights the sequences for which HMMSTUFF can predict the structure by homology, identifying those that meet the criteria for reliable structural prediction. (C) Shows the closest known structure for each sequence, with black X's representing sequences with newly solved cryoEM structures that were not used in the development of HMMSTUFF. (D) Compares the structure of one of these sequences (FOR103 from Karimi-Farsijani *et al.* 2024) with its newly solved cryoEM structure (PDB ID 9EME) against the structure inferred by homology using HMMSTUFF.

The plots in Fig. 2A–C were generated using the t-distributed Stochastic Neighbor Embedding (t-SNE) implementation in scikit-learn. For each sequence in our dataset, we calculated scores with all six previously trained models. These scores were used as features, creating a 6D feature vector for each sequence. We then applied t-SNE to reduce these 6D vectors to two dimensions, allowing for visualization in the plots shown in Fig. 2A–C.

## 3 Results

### 3.1 Distribution of the known LC amyloid sequences

In this paper, one of our key objectives is to examine the distribution of known LC amyloid sequences and determine the fraction of these sequences for which a suitable structural template exists. To address the first question, we scored each sequence available in our dataset to the six available template structures using HMMSTUFF and collected the resulting scores. We then applied t-distributed stochastic neighbor embedding (TSNE) to reduce the 6D data to two dimensions, allowing us to visualize the distribution of these sequences. While TSNE is a commonly used method, it is important to note that this analysis is intended purely for visualization, as distances in the original 6D space may not be accurately represented in the reduced 2D space.

Figure 2A presents a plot of the known LC amyloid sequences, categorized by their germline class. The data reveals three distinct clusters, with Lambda VL6 forming its own separate cluster.

In addition to analyzing sequence distribution, we wanted to determine the proportion of sequences for which we can confidently predict amyloid conformation. To make such predictions, we selected LC sequences that aligned without gaps for at least 80% of the template length (using the alignment generated by HMMSTUFF) and had a HMMSTUFF score of at least 0.5 for at least one of the possible templates. The requirement for high coverage and ungapped regions is crucial, as we cannot predict amyloid structures *de novo* and we have to rely on homology-based modeling. Such a method makes it almost impossible to effectively assign the backbone gap regions in understudied structures such as amyloids. Moreover, the interactions that stabilize amyloid structures are challenging to predict, so high sequence identity is necessary to reduce the likelihood of errors in estimating the forces driving conformational stability.

Our analysis found that only 27.5% of the sequences in our dataset meet the criteria necessary for reliable homology-based modeling. Figure 2B provides a visual representation of the sequences that are currently amenable to structural modeling. There are entire clusters of sequences for which we have absolutely no structural information, and that would strongly benefit by structural experimental investigation. Germlines such as Kappa K1, and K4 do not have any viable structural template at the moment. Experimental investigation of the structures of some of these LC amyloids would provide extremely valuable information about the connection between LC sequences and LC amyloid conformation.

### 3.2 Newly solved LC amyloid structures

In a recent paper (Karimi-Farsijani *et al.* 2024), the authors solved the cryo-EM structure of two new LC amyloids. These proteins were not included in our initial analysis, making them valuable for assessing the behavior of HMMSTUFF in real-world applications. In Fig. 2B and C, we included the scores of these two sequences, highlighting them with an “X.”

Interestingly, FOR110, which is reported to be very different from the other LC amyloid structures, is positioned by HMMSTUFF within a cluster of sequences lacking a known structural template in the TSNE plot. For this sequence, HMMSTUFF is unable to identify any viable template, with a maximum score of 0.14 for the template 6Z1O, which is significantly lower than the required threshold of 0.5. In contrast, FOR103 is identified by HMMSTUFF as homologous to the 6Z1O template. Figure 2D compares the experimentally solved structure of FOR103 with the homology model generated by HMMSTUFF.

### 3.3 HMMSTUFF on AlBase data

**Figure 3.**
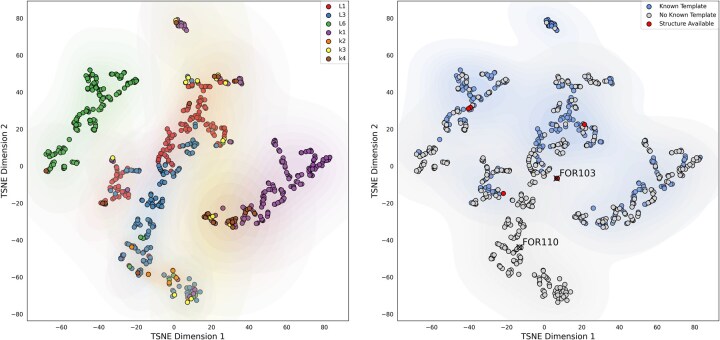
Analysis of known LC amyloid sequences obtained from ALBase using HMMSTUFF. The figure replicates the analysis from Fig. 2, using sequences from the ALBase database. The left plot displays the t-SNE dimensionality reduction of the sequences, color-coded by germline. The right plot shows the portion of the sequences that can be modeled by HMMSTUFF, with FOR103 and FOR110, two sequences with newly available CryoEM structures, highlighted for reference.

Our dataset is manually curated and it contains only sequences that have been recently identified in living patients. There are, however, other resources that contain information about AL sequences. One of these is the ALBase database. We therefore built an additional dataset from this resource. We downloaded 889 sequences reported to form AL in vivo and we performed the same analysis we did in Fig. 2. Such analysis is provided in Fig. 3.

From the analysis, we excluded all sequences for which HMMSTUFF returned a log probability of -infinity for at least one of the templates, indicating a near-zero probability for those sequences. In this refined dataset, we observe results similar to those from our own dataset, with most germlines clustering together.

### 3.4 Limitation of the model

HMMSTUFF is not intended as a long-term solution for analyzing and predicting the structure of LC amyloids and the user should be fully aware of its intrinsic limitations.

First, as mentioned earlier, HMMSTUFF can only handle a limited subset of sequences. The tool was designed as a temporary aid for investigating light chain amyloidosis, targeting sequences with highly similar structural templates available and therefore it can only address a small fraction of LC amyloids. We chose this approach due to the limited data available in the field, which makes developing a robust predictive model unfeasible. Current knowledge and experimental data are insufficient to support the creation of a reliable machine learning-based predictor. Additionally, with only six known structures, likely not representative of the entire conformational space, even proper validation is not achievable. For this reason, we decided to focus solely on cases where the solution is nearly self-evident. Still, this temporary solution can serve as a guide for experimental studies, helping to maximize the insights gained from them.

A second limitation lies in our incomplete understanding of how sequence similarity correlates with structural similarity in LC amyloids. Additionally, amyloids are intrinsically polymorphic, with their structure determined by a complex interplay between sequence and environmental conditions. This means that even identical sequences can adopt different structures depending on the surrounding context. While experimental evidence suggests that highly similar LC amyloid sequences tend to form similar structures in similar conditions, this correlation becomes uncertain at lower levels of sequence identity. Further experimental data and structural analyses are necessary to address this gap. To mitigate this issue, we took a conservative approach, limiting the usability of the tool to cases with very high sequence similarity.

Lastly, the model uses force fields for side chain optimization. Although these methods have shown promise in handling amyloid structures to some extent (Louros *et al.* 2020), their performance with LC amyloids remains unverified. Additional structural data and stability experiments will be essential to fully evaluate their effectiveness. Finally, we would like to stress that, given the current state of the field, any quantitative performance evaluation based on the limited structural data available is likely to be heavily biased and unreliable.

## 4 Conclusions

As mentioned above, most of the LC amyloid space remains unexplored from both a sequence and structural perspective. To date, only six structures have been experimentally solved, and these only represent λ1, λ3, and λ6 germlines. For example, none of the kappa class structures have been resolved. HMMSTUFF has the potential to model various kappa sequences, expanding the structural coverage to some κ1, κ3, and κ4 sequence, but the accuracy of these predictions should be approached with caution due to the limited experimental data. The germlines with good experimental coverage and for which HMMSTUFF can provide clues about their conformation are λ1 and λ6 germlines, while the ones which are left behind, even by HMMSTUFF, are a large amount of the κ and the totality of the κ2 subtypes.

HMMSTUFF, however, is not a long term solution to the problem but rather a way to make the most of the limited data currently available. It acts as a temporary measure, leveraging existing information without replacing the need for new experimental data, both from sequence and structural perspectives. Without additional data, it is highly unlikely that we can develop more reliable mathematical models to accurately predict LC amyloid structures. Therefore, the goal of HMMSTUFF is not to solve the issue of LC amyloid structure prediction but to guide experimental efforts toward investigating the most valuable amyloids for expanding our understanding of LC amyloids. Our goal is to support researchers by helping them prioritize LC amyloids most likely to provide valuable insights through structure-based experiments, such as cryoEM, thereby expanding our understanding of the relationship between LC sequences and structures.

Conflict of interest: None declared.

## Data Availability

All the data related to this article is available at https://github.com/grogdrinker/hmmstuff.
